# Declaration of local chemical eradication of the Argentine ant: Bayesian estimation with a multinomial-mixture model

**DOI:** 10.1038/s41598-017-03516-z

**Published:** 2017-06-13

**Authors:** Yoshiko Sakamoto, Naoki H. Kumagai, Koichi Goka

**Affiliations:** National Institute for Environmental Studies, Onogawa 16-2, Tsukuba, Ibaraki 305-8506 Japan

## Abstract

Determining the success of eradication of an invasive species requires a way to decide when its risk of reoccurrence has become acceptably low. In Japan, the area populated by the Argentine ant, *Linepithema humile* (Mayr), is expanding, and eradication via chemical treatment is ongoing at various locations. One such program in Tokyo was apparently successful, because the ant population decreased to undetectable levels within a short time. However, construction of a population model for management purposes was difficult because the probability of detecting ants decreases rapidly as the population collapses. To predict the time when the ant was eradicated, we developed a multinomial-mixture model for chemical eradication based on monthly trapping data and the history of pesticide applications. We decided when to declare that eradication had been successful by considering both ‘eradication’ times, which we associated with eradication probabilities of 95% and 99%, and an optimal stopping time based on a ‘minimum expected economic cost’ that considered the possibility that surveys were stopped too soon. By applying these criteria, we retroactively declared that Argentine ants had been eradicated 38–42 months after the start of treatments (16–17 months after the last sighting).

## Introduction

Biological invasions are environmental problems globally: they seriously impact biodiversity and disrupt ecosystem processes^1–3^. Many programs conducted to eradicate invasive species have eventually proven to be unsuccessful because eradication is a challenging task^4,5^. Eradication is difficult for two reasons^6^. First, it is hard to reduce a population to the point of extinction. Second, it is difficult to confirm that eradication has succeeded with imperfect monitoring surveys. Economic considerations limit how long monitoring surveys can be conducted. Management should stop at some point when surveys continue to indicate the absence of an invasive species. However, the risk that the invasive species is still present can be high, and in the absence of eradication, the population of the invader may rebound^7,8^. When monitoring stops is the most important determinant of eradication success.

Ant species have been highly successful invasive taxa worldwide^9^. Many programs to eradicate invasive ants have failed because of inability to control further dispersal^10–12^. Consequently, only 10–15 cases of successful eradications of invasive ants have been documented over the past century of eradication efforts^12–14^. In the absence of standard protocols for determining eradication success^15^, Hoffmann, *et al*.^12–14,16^ have argued that eradication of an invasive ant is indicated by the absence of the target invasive ant species for two years (24 months) after treatment because two reproductive events have passed within that timeframe. This criterion has been used in most publications related to ant eradications. However, the life cycles of invasive ants differ between species, and the frequency of monitoring surveys has varied between eradication programs. Moreover, the two-year criterion is not statistically supported. To establish an unequivocal criterion is difficult, because there have been few reports of successful eradications. Conversely, the absence of a criterion with a sound statistical basis may lead to eradication failure.

This problem is currently being considered as a part of the program to eradicate the Argentine ant, *Linepithema humile* (Mayr), in Japan. In Japan, a list of “specified invasive alien species” appears in the 2005 law, ‘Invasive Alien Species Act’, according to which the specified invasive alien species must be eradicated if it invades Japan. The list designates four ant species. One of them, the Argentine ant, was introduced into Japan within the last 20 years^17^. Programs to eradicate the ant have been conducted in some areas by local governments in accord with the *Manual for Control of Argentine Ants*^18^, produced by the Ministry of Environment. In Tokyo, there are two test sites where Argentine ants were found in 2010, and those sites have been treated with a pesticide since April 2011. Monitoring surveys at those sites are being continued every month, although Argentine ants are no longer seen, and treatments have already been terminated. In such situations, there is a need to predict the probability that the population has been completely eradicated.

Failure to detect a focal species does not always mean that the species has been successfully eradicated. Because the probability of detection is <100% when the species is present, non-detection of a species can occur either because the species is absent or because the species is present but is not detected. Statistically rigorous methods have recently been developed to estimate the presence of a population or community using the detection frequency of the species based on data from repeated observations^19,20^. Among these models, the multinomial-mixture model^21^ can flexibly incorporate the influence of non-detection and estimate abundance based on fluctuating counts among repeated observations. Specifically, the “removal protocol” of the multinomial-mixture model can use the decline in the detected abundance of the focal species (i.e., right-censored data) in response to eradication management to estimate latent abundance, including individuals that have not yet been observed. This model is well suited for estimating the probability of eradication of Argentine ants, because the abundance of Argentine ants fluctuates greatly and often decreases to non-detection levels in response to eradication management within a timeframe that is short (several months) compared to the longevity of worker ants (one-half to one year) (The Supplementary Note summarizes fundamental knowledge about the biology of the Argentine ant). The original multinomial-mixture model incorporated detection uncertainty associated with repeated removals of individuals, but the decrease of the ant population was due to both repeated removals and eradication efforts with chemicals. Therefore, we needed to consider two sources of uncertainty, one associated with detection and the other with eradication.

In this study, we developed a new version of the multinomial-mixture model for chemical eradication that incorporates changes in detection probability associated with removals by chemical eradication and by capture samplings of ants via sticky traps. We then applied this model to provide criteria for estimating the probability of successful eradication of this invasive ant. We also compared the estimates of the original multinomial-mixture model and those of our new version of that model. We specifically wanted to know how much time elapsed between the start of the program and the time when it could be safely declared that the invasive ant had been eradicated (‘eradication’ time). This time was based on probabilities of either 5% or 1% that ants were still present. Furthermore, we determined an optimal stopping time based on a ‘Minimum expected cost’ model, a trade-off between the cost of continuing the monitoring survey and the eventual cost of escape if the survey were stopped too soon^6,7^.

## Results

### Estimation of eradication time

**Figure 1:**
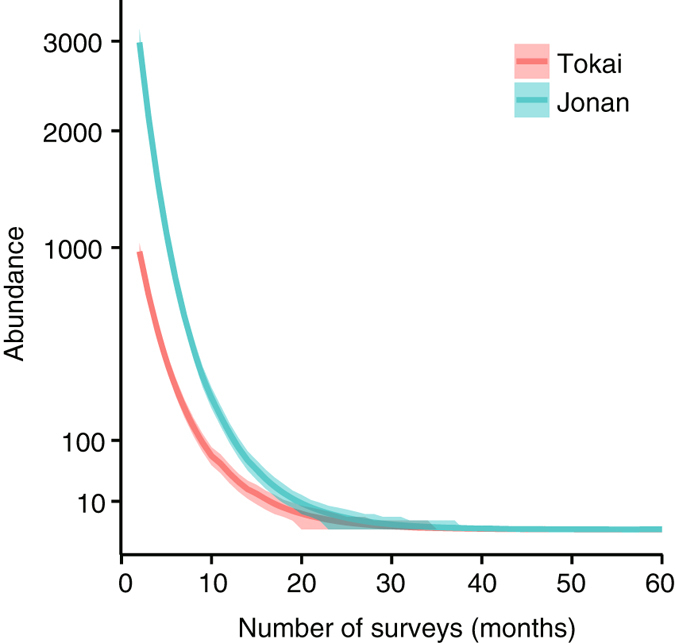
Changes in the estimated number of Argentine ants with the number of surveys. Dark-coloured curves indicate posterior means. Shading indicates the 95% credible intervals.

**Figure 2:**
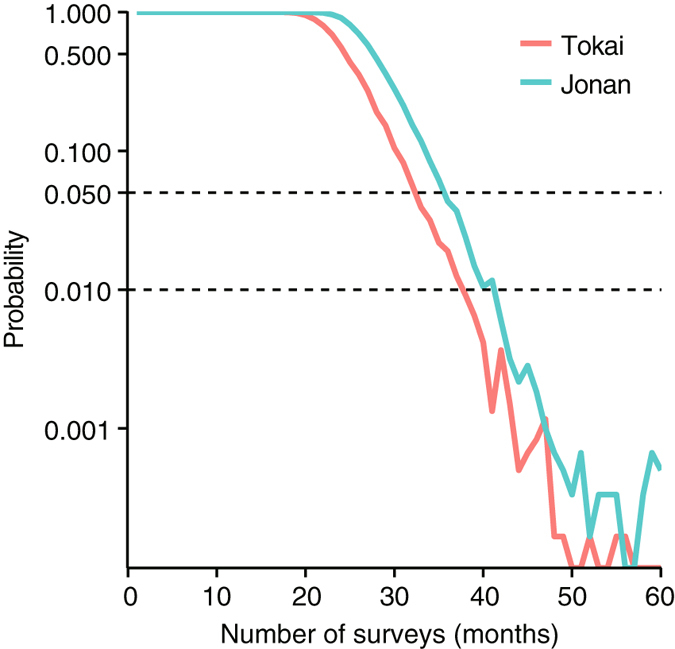
Posterior probabilities of the estimated occurrence of Argentine ants versus the number of surveys at the Tokai and Jonan sites. Dashed lines indicate posterior probabilities of 0.01 and 0.05.

The observations failed to detect ant workers by the 21st and 26th months after the start of the eradication program at the Tokai and Jonan sites, respectively. Altogether, 1164 and 3565 individuals were captured throughout the 59 consecutive months of surveys at Tokai and Jonan, respectively. By applying the multinomial-mixture model for chemical eradication to the patterns of the observed abundances, we estimated the per capita probabilities of detection by a sticky trap and of death by pesticide application to be 0.279 [95% credible interval (CI): 0.268, 0.287] and 5.39 × 10^–3^ [1.28 × 10^–4^, 1.93 × 10^–2^], respectively (cf., the detection probability was 0.287 [0.276, 0.289] when estimated by the original multinomial-mixture model, which considers only the uncertainty due to capture samplings). Based on the per capita probabilities of detection and death, we obtained the joint probability of an individual’s not being removed during any previous survey and being removed in the focal survey. We then derived the estimated changes of the number of ants found at each sampling (see Methods). The initial numbers of ants before the start of the eradication program were estimated to be ca. 1400 and 4200 at Tokai and Jonan, respectively. The number of survivors declined to nearly undetectable levels after 20 months at each site (Fig. 1). The estimated probability that ants were still present then declined to 1% (5%) in month 38 (33) at the Tokai site and in month 42 (36) at the Jonan site (Table 1) (Fig. 2). It therefore took 12 months at the Tokai site and 10 months at the Jonan site after the last observations for the probability of eradication to reach 95%, and it took 17 months at the Tokai site and 16 months at the Jonan site for the probability of eradication to reach 99%. Compared to the eradication times estimated with the original multinomial-mixture model, the 99% eradication time at Jonan was one more month longer but did not differ at Tokai.

**Table 1:** Month and time details in the eradication program of Argentine ants.

	Tokai		Jonan	
	months	time	months	time
Start of administering pesticide		Apr 2011		Apr 2011 / Mar 2012*
Maximum period of detecting the ant	21	Dec 2012	26	May 2013
Last observation of the ant		Dec 2012		May 2013/Dec 2013*
95% eradication for chemical control	33	Dec 2013	36	Feb 2015*
99% eradication for chemical control	38	May 2014	42	Aug 2015*
95% eradication in original	33	Dec 2013	36	Feb 2015*
99% eradication in original	38	May 2014	41	Jul 2015*
Optiimal duration for minimum expected cost	33	Dec 2013	36	Feb 2015*

*A plot of Jonan (Plot I) where administering pesticide was late due to another experiment.

### Minimum expected cost

**Figure 3:**
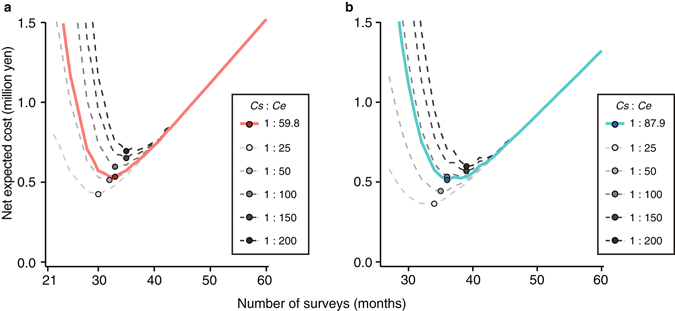
The net expected cost of declaring eradication versus the number of months after the last ant sighting (solid coloured curves) if management continued as usual, but no more ants were detected. The current monthly cost of management (*Cs*) is JPY 40,000, and we assumed that the costs of declaring eradication were 59.8 and 87.9 times the monthly monitoring cost at (**a**) Tokai and (**b**) Jonan, respectively. The dotted lines indicate results for 5 different cost ratios.

We calculated the net expected cost (NEC) after the last sighting as a function of the estimated probability of ants still being present, given the monthly cost of management (*Cs* = JPY 40,000) and the cost of escape and damage (*Ce* = 59.8*Cs* and 87.9*Cs* for Tokai and Jonan, respectively). As the number of months without detection increases, the NEC initially decreases but then increases again as the monthly costs of monitoring (*Cs*) accumulate (Fig. 3). The minimum point on each curve is the optimal survey time that minimizes the NEC. The optimal duration of no detection of worker ants before stopping the monitoring survey was 33 months from the start of the program at Tokai and 36 months at Jonan (Fig. 3); these times coincided with the 95% eradication times at both sites. The NEC of monitoring for the optimal survey time was JPY 534,000 at Tokai and JPY 512,000 at Jonan. The NECs corresponding to a 99% probability of eradication were JPY 662,000 at Tokai and JPY 621,000 at Jonan, 24.0% and 21.2% higher, respectively, than the minimum NEC.

If the cost of escape and damage (*Ce*) were lower or higher than our baseline assumption, the optimal time to continue monitoring would be shorter or longer, respectively. If the cost of declaring eradication prematurely were 25–200 times the monthly cost of monitoring, the optimal time for monitoring would be 30–35 months at Tokai and 34–39 months at Jonan (Fig. 3).

## Discussion

We developed a multinomial-mixture model for chemical eradication of a pest to predict when the probability of detection of the pest was below a specified level. Application of this model did not change the times associated with the 95% and 99% probabilities of eradication of Argentine ants from the times estimated with the original multinomial-mixture model because the estimated death probability for chemical eradication was low. The reasons for the low estimated death probability associated with the pesticide, despite the high efficacy of the pesticide in eliminating ants^22^, are as follows. First, the pesticide effect may have been underestimated because data from May (the second sampling) were used. April, which is when the eradication program was started, is considered to be the most effective season for pesticide use because April is the time when reproductive members of the population develop (Data for April 2011 were not used because most of the traps were lost during that survey. See Methods). Second, the *per capita* death probability *d* is expected to be very high at the first application of a pesticide, and if it is 0.9, the joint probability of death at the second survey is (1 − 0.9) × 0.9 = 0.09, the indication being that there is a steep decrease in death probability after the first application. In any case, because the credible interval of the death probability was large compared to the mean value, taking account of the variation in death probability helped to reduce the uncertainty in the predicted time of eradication, a goal of this study.

Resources should be invested in eradication of a target invasive species in a way that provides the greatest benefit^7,23^. Based on this philosophy, it would be better to invest funding to offset the eventual cost of escape rather than to pay for monitoring surveys. When the NEC estimation is applied to the Argentine ant eradication program, the indication is that the optimal time to stop monitoring would precede the eradication times (99% probability of eradication success) by 5 months at Tokai and by 6 months at Jonan if the population rebounds to the level before the start of the eradication program (Fig. 3). On the one hand, the curves (Fig. 3) show that a too-early declaration of eradication is associated with a high cost of possible escape and damage. On the other hand, the increase of the NEC after the optimal stopping time was small compared to the magnitude of the eradication cost because the probability of detection decreased dramatically with increasing numbers of surveys, and the cost of the monitoring surveys was low. Actually, the cost at the 99% eradication time was not more than 1.5 times the minimum NEC. In another case of invasive species, foxes on Phillip Island, Australia, the dynamics of the NEC caused the monitoring cost to accumulate rapidly after the minimum^6^, unlike our case of Argentine ants. However, those estimates of the cost of eradicating the pest were calculated when the population was assumed to have rebounded to levels seen before the eradication program. In fact, the cost of escape and damage is difficult to estimate accurately. Argentine ants forgo dispersal through nuptial flights. However, because they can disperse by human-mediated long-distance transport^24–26^, their population growth is not accurately estimated. There is a risk that the population could exceed the population at the start of the eradication program. The NEC dynamics in this study suggest that continued monitoring is financially advisable, even if the monitoring survey continues for a long time as a precaution.

Given the difficulty of predicting the cost of escape and the low cost of the monitoring survey, the theoretical probability of detection suggests that monitoring until the 99% eradication time is advisable. We therefore retroactively declared that eradication of Argentine ants occurred at the Tokai site in May 2014 (17 months after the last sighting) and at the Jonan site in August 2015 (16 months after the last sighting). Because the life cycle of Argentine ants is characterized by a single reproductive event per year^27^, a 24-month monitoring period is needed post treatment to satisfy the criterion that two reproductive events have passed^12–14^. The implication of our study is that the time needed to ensure eradication is shorter than this criterion. However, what matters is not just the period of time, but the frequency and number of surveys. Monitoring surveys have been conducted at a frequency of once or twice per year in almost all previous studies^12,13^, in contrast to our monthly surveys. If the frequency of our monitoring surveys had been reduced to decrease the cost, the detection error would have become much larger. Consequently, the probability of eradication success would have been difficult to estimate precisely. After successful eradication, periodic monitoring (e.g., once per year) in ports would allow for early detection of reinvasion of new alien ants or populations, because port areas are at high risk for invasive ants^28^.

In conclusion, we have provided criteria for determining the time required for eradication of an invasive ant species based on a statistical analysis that took into consideration probabilities and costs. The multinomial-mixture model for chemical eradication that we developed can be applied to cases where it is difficult to estimate population growth due to ecological characteristics, such as the formation of colonies, or to cases where there is not enough time to obtain population parameters because pesticides are quite effective and the pest abundance declines to undetectable levels within several months. The multinomial-mixture model is effective even subject to these constraints.

## Methods

### The eradication program

We now illustrate the application of the model to a case study of the program to eradicate Argentine ants from Tokyo, Japan^22^. There were two sites, Tokai and Jonan. These sites are located on landfill islands in Tokyo Bay, 2 km apart from each other, and were infested by Argentine ants. Areas of approximately 8.5 and 16 ha for chemical treatment were established at the Tokai and Jonan sites, respectively. The areas were divided into approximately 1–ha plots along streets. The program was conducted once per month from April 2011 to March 2016, except in a plot where it was started in March 2012 (Table 1). A toxic bait paste (0.005% fipronil) (Fumakilla, Ltd., Hiroshima, Japan) was placed at intervals of 5–10 m along the streets and buildings. Whether bait paste should be applied in a given month was decided on the basis of monitoring during the previous months; bait paste was applied if there was a finding of Argentine ants in the same plot during the previous six months, and application of paste was stopped if no Argentine ant was found for approximately six consecutive months. When we found broods or queens in shrubberies or under concrete blocks during bait paste application, we sprayed into them a toxic liquid, 0.005% fipronil (Fumakilla, Ltd.). Within plots, a sticky trap (Monitoring PP Trap #J, Kankyokiki Co., Ltd., Osaka, Japan: 8.8 × 19.5 × 2.2 cm) was placed approximately every 50 m along the streets and buildings to detect ants and to evaluate their abundance once a month. The traps were collected 3 days after they were set out. The number of worker ants collected was determined in the laboratory. Visual observations were conducted by walking for 20–30 minutes per hectare at the same time that the traps were set out at each site as a precaution to be sure that any ants present were detected. Detection probability was higher with the trapping method than with visual observations (Sakamoto *et al*., unpublished).

### Description of model for estimating eradication time

We developed a “removal protocol” for a multinomial-negative binomial mixture model^21^ that could incorporate not only detection probability by physical samplings but also death probability by pesticide applications. We regarded the individuals physically captured by sticky traps as those detected by physical samplings, and the others as being removed by pesticide applications including bait paste and spraying. Note that there are no distinct differences between the individuals in aboveground and underground environments (See details in ecological information in Supplementary Note). In the base model, only the joint probability of an individual’s being removed in the course of capture samplings was considered. The probability of an individual’s being captured in the first sampling at point *i* is *π*_*1,i*_ = *c*_*1,i*_, and that of not being captured in the first sampling and being captured in the second sampling is *π*_*2,i*_ = (1 − *c*_*1,i*_) *c*_*2,i*_, where *c*_*t,i*_ is the *per capita* detection probability via capture sampling at time *t* at point *i*. Thus, the joint probability of an individual’s not being captured until the *t*–1-th sampling and being captured in the *t*-th sampling at point *i* is *π*_*t,i*_ = (1 − *c*_*1,i*_) (1 − *c*_*2,i*_) ···  (1 − *c*_*t-1,i*_) *c*_*t,i*_.

In the model, we included the joint probability of an individual’s being killed via contact with toxic liquid and/or paste. The death of an ant via the pesticide can be modelled in the same way as the probability of its detection in sticky traps, because individual ants were killed by direct contact with the pesticide or by secondary contact with it brought back to the nest by other ants. In our model, the probability of an individual’s being caught in a sticky trap without being killed by bait paste in the first sampling at point *i* is *π′*_*1,i*_ = (1 − *d*_*1,i*_) *c*_*1,i*_, and that of an individual’s not being killed by the pesticide nor caught in a sticky trap in the first sampling and being caught in a sticky trap in the second sampling is *π′*_*2,i*_ = (1 − *d*_*1,i*_) (1 − *c*_*1,i*_) (1 − *d*_*2,i*_) *c*_*2,i*_, where *d*_*t,i*_ is the *per capita* probability of death via bait paste at time *t* at point *i*. An ant that contacted the pesticide directly or indirectly is expected to die within the monthly three-day sampling period because the bait paste had disappeared by the end of each three-day sampling period. According to this logic, the number of ants removed, *y*_*t,i*_, in the *t*-th sampling at point *i* is expressed as:

1 $${y}_{t,i} \sim {\rm{Multinomial}}(\pi {^{\prime} }_{t,i}/{\Sigma }_{k}\pi {^{\prime} }_{k,i},{n}_{i})$$

where *n*_*i*_ = Σ_*k*_ *y*_*k,i*_ denotes the total number of ants removed at point *i* during all samplings throughout the study at that point. *n*_*i*_ is linked to a latent variable, *N*_*i*_:

2 $${n}_{i} \sim {\rm{Binomial}}({\Sigma }_{k}\pi {^{\prime} }_{k,i},{N}_{i})$$

where *N*_*i*_ is the number of individuals that inhabited point *i* at the beginning of the surveys. In Equation 2, *n*_*i*_ is the sum of all ants removed from *N*_*i*_ during all surveys. This link between *n*_*i*_ and *N*_*i*_ allows stochastic variation in their relationship. Furthermore, because *N*_*i*_ is anticipated to be highly variable, *N*_*i*_ is approximated with a negative binomial distribution as follows:

3 $${N}_{i} \sim {\rm{NegBin}}(\mu ,\sigma ),$$

with variance $$\mu +{\mu }^{2}/\sigma $$.

Finally, we derived the Bayesian posterior distribution of the expected number of surviving ants at each site (*i*) at each point in time (*t*), *s*_*t,i*_, using the joint probability of an individual’s being neither killed nor captured until the *t*-th sampling, *ϕ*_*t,i*_:

4 $${s}_{t,i} \sim {\rm{Bin}}({\varphi }_{t,i},{N}_{i}).$$

where ${\varphi }_{t,i}={{\rm{\Pi }}}_{k}(1-{d}_{k,i})(1-{c}_{k,i})$ was based on the posteriors of *d*_*k,i*_ and *c*_*k,i*_, obtained above.

Further, we calculated the total number of survivors at time *t* as *S*_*t*_ = Σ_*j*_ *s*_*t,j*_. Then, we derived the expected probability that an ant was still alive at each time *t* at each site (*i*) as *Ps*_*t*_ = Pr(*S*_*t*_ < 1). Here, we applied the 95% and 99% credible intervals of *Ps*_*t*_ as the index of eradication success. Furthermore, we defined the month after which *Ps*_*t*_ became less than 0.05 and 0.01 at a site (i.e., site level probability) as the time to declare that eradication of Argentine ants at the site was 95% and 99% probable, respectively.

### Model application

We modelled the decline of Argentine ant workers as a result of repeated removals during the consecutive 59 months that samples were collected at the 23 and 28 points at Tokai and Jonan, respectively. Data for April 2011 (the first sampling) were not used, because we deployed sampling traps that were not secured to the ground and consequently lost most of the traps. First, we considered the possibility that a time-specific covariate might have influenced the detection probability:

5 $$c{^{\prime} }_{t,i}=1-\exp (-\exp ({b}_{0}+{b}_{1}\cdot Temperatur{e}_{t}))$$

where the *β*_*k*_ (*k* = 0–1) are linear predictor parameters, which are to be estimated. The parameter *Temperature*_*t*_, the monthly mean of the air temperature in Haneda, Tokyo^29^ during the *t*-th sampling month, was the covariate of detection probability, because air temperature is expected to affect the activity of ants^30^ and therefore may increase detection rate. However, we found that the estimated temperature coefficient was not significant (*β*_*1*_: 9.11 × 10^–4^ [95% CI: −5.27 × 10^–3^, 3.40 × 10^–3^]). Thus, we assumed that the probability was independent of temperature.

We also applied a correction for the influence of losses of some traps during the samplings as follows:

6 $${c}_{t,i}=c^{\prime} \times hol{d}_{t,i}$$

where *hold*_*t,i*_ denotes whether a trap at point *i* remained in place during sampling at time *t* and had values of 1 (held) or 0 (lost). By multiplying *c′* by *hold*_*t*,*i*_, detection rates for lost traps were corrected to zero. Similarly, we assumed the death probability from contact with bait paste to be a constant and corrected it for discontinuation of bait paste applications:

7 $${d}_{t,i}=d^{\prime} \times che{m}_{t,i}$$

where *chem*_*t,i*_ denotes a binary variable that indicates whether the bait paste was applied at a sampling time and took values of 1 (applied) or 0 (discontinued).

The modelling was performed using the Bayesian inference in WinBUGS 1.4.3. The WinBUGS code is included in the Supplementary Dataset. We used nearly non-informative prior distributions for the parameters. Priors for the probabilities of detection and death were specified as uniform distribution priors between 0 and 1. Priors for μ and σ in Equation 3 were specified using the gamma distribution. We derived Bayesian estimates from 220,000 Markov Chain Monte Carlo iterations after a 20,000-iteration burn-in and thinning at intervals of 100. The R-hat values of estimated parameters, which ranged between 1.00 and 1.01, implied that convergence had been achieved. We used three chains to confirm that estimated values were independent of initial parameters.

### Minimum expected cost

The optimal time to stop monitoring and declare eradication successful was supported not only by the 99% probability of eradication success but also by cost management. We also calculated the NEC of declaring the ant eradicated for the *m* months since the last sighting^6,7^:

8 $${\rm{NEC}}(m)=(m-1)\cdot Cs+[1-P({N}_{T}\le 1|m)]\cdot Ce,$$

where *Cs* is the monthly cost of management, and 1 − *P*(*N*_*T*_ ≤ 1|*m*) is the probability that the ant is still present despite *m* consecutive months without detection. Therefore, we used the estimated probability of the ants being present, *Ps*_*t*_, at each of the Jonan and Tokai sites. *Ce* is the cost of declaring ants eradicated, even though they are still present. The optimal month to declare eradication was the month in which the expected cost was lowest^6,7^.

We substituted plausible values for *Cs* and *Ce* based on the actual expenses of the program. The cost of monitoring, *Cs*, at both sites could be calculated by using the mean number of person-hours and consumables needed and was found to be approximately JPY 40,000 per month at each site. For the six months after the last sighting, additional pesticide applications (approximately JPY 10,000 per month) were also needed. The cost of escape and damage, *Ce*, was the expected cost if the ant was still present after being declared eradicated and if the ant population was able to rebound to levels that were present before the eradication program after the monitoring survey was stopped. We calculated the cost when monitoring stopped at a time corresponding to 99% eradication probability using the above information and actual manpower and pesticide costs. The total costs were JPY 2,393,969 and 3,517,071 (*Ce* = 59.8*Cs* and 87.9*Cs*) over the previous 38 months and 42 months at Tokai and Jonan, respectively. We used these costs as the baseline cost estimates for declaring eradication (*Ce* = 59.8*Cs* and 87.9*Cs* for Tokai and Jonan, respectively). However, although the future expected cost of the consequences of declaring eradication prematurely is uncertain, we have provided a range of possible costs to estimate the optimal time (*Cs*:*Ce* *=* 1:25 to 1:200).

## Electronic supplementary material

Supplementary information

Supplementary data

Program_code
