# Author Correction: Declaration of local chemical eradication of the Argentine ant: Bayesian estimation with a multinomial-mixture model

**DOI:** 10.1038/s41598-018-24984-x

**Published:** 2018-04-24

**Authors:** Yoshiko Sakamoto, Naoki H. Kumagai, Koichi Goka

**Affiliations:** National Institute for Environmental Studies, Onogawa 16-2, Tsukuba, Ibaraki 305-8506 Japan

Correction to: *Scientific Reports* 10.1038/s41598-017-03516-z, published online 13 June 2017

The original Supplementary data file published with this Article contained a typographical error where ‘toxi’ now reads ‘chem’. The correct Supplementary data file now accompanies the Article.

