# Supplementary material for: Declaration of local chemical eradication of the Argentine ant: Bayesian estimation with a multinomial-mixture model
Source: Sci Rep. 2017 Jun 13;7:3389. doi: 10.1038/s41598-017-03516-z (PMC5469785; doi:10.1038/s41598-017-03516-z)
Supplement: Supplementary file 1 — Supplementary information [file 41598_2017_3516_MOESM1_ESM.doc]

Supplementary Note: Ecological information about Argentine ants

Declaration of local chemical eradication of the Argentine ant: Bayesian estimation with a multinomial-mixture model

Yoshiko Sakamoto^*,†^, Naoki H. Kumagai ^†^ & Koichi Goka

National Institute for Environmental Studies, Tsukuba, Ibaraki 305-0053, Japan

^*^Corresponding author

email: [sakamoto.yoshiko@nies.go.jp](mailto:sakamoto.yoshiko@nies.go.jp)

Tel/Fax: +81-(0)29-850-2480

^†^These authors contributed equally to this work.

**Ecological information about Argentine ants**

Argentine ants are polygynous; colonies comprise up to 16.3 queens per 1000 workers, but the ratio of queens to workers varies seasonally ^1^. Argentine ant queens mate with males intranidally once a year within the nest. Colonies spread on the ground via budding; they do not disperse via nuptial flight ^2^. A mated queen produces 20–30 eggs per day ^3^. The mean period from egg to adult is approximately 70 days at 28 °C ^4^. The longevities of queen and worker adult are approximately 10 months ^1^and 6–12 months ^2^, respectively, which are relatively short compared to other ant species. In general, young ants care for the brood, and old ants forage in accord with the division of labour ^5^ ^6^. The foraging range of an Argentine ant worker is large and may exceed 60 m from the nest ^7^. Argentine ants form supercolonies, in which there are no colony boundaries and no intraspecific aggression; workers can therefore contribute to accelerating colony growth instead of fighting ^8^.

**Effectiveness of fipronil products on Argentine ants**

Fipronil is a member of the phenyl pyrazole class of pesticides and is a potent disrupter of the arthropod central nervous system via interference through the gamma-aminobutyric acid- (GABA-) regulated chloride channel ^9^. The fipronil baits used in our study attract Argentine ants and other ant species. Foragers take a piece of bait to their nest and share the bait through trophallaxis with other colony members; therefore not all workers have to visit baits for effective control of ant colonies ^10^. Directed spray of fipronil is also effective in controlling other colony members because fipronil is transferred from exposed ants to unexposed ants via grooming or necrophoresis ^11^.

**References**

1 Keller, L., Passera, L. & Suzzoni, J. P. Queen execution in the Argentine ant, *Iridomyrmex humilis*. *Physiol Entomol* **14**, 157-163 (1989).

2 Newell, W. & Barber, T. C. The Argentine ant. *Bur. Entomol. Bull.* **122**, 1-98 (1913).

3 Abril, S., Oliveras, J. & Gomez, C. Effect of temperature on the oviposition rate of Argentine ant queens (*Linepithema humile* Mayr) under monogynous and polygynous experimental conditions. *J Insect Physiol* **54**, 265-272 (2008).

4 Abril, S., Oliveras, J. & Gomez, C. Effect of temperature on the development and survival of the Argentine ant, *Linepithema humile*. *J Insect Sci* **10**, 97 (2010).

5 Odonnell, S. & Jeanne, R. L. Implications of senescence patterns for the evolution of age polyethism in eusocial insects. *Behav Ecol* **6**, 269-273 (1995).

6 Beshers, S. N. & Fewell, J. H. Models of division of labor in social insects. *Annu Rev Entomol* **46**, 413-440 (2001).

7 Vega, S. Y. & Rust, M. K. Determining the foraging range and origin of resurgence after treatment of Argentine ant (Hymenoptera: Formicidae) in urban areas. *J Econ Entomol* **96**, 844-849 (2003).

8 Holway, D. A., Suarez, A. V. & Case, T. J. Loss of intraspecific aggression in the success of a widespread invasive social insect. *Science* **282**, 949-952 (1998).

9 Rhône Poulenc. *‘Fipronil’ worldwide technical bulletin* (Agrochimie, 1996).

10 Knight, R. L. & Rust, M. K. Efficacy of formulated baits for control of argentine ant (Hymenoptera, Formicidae). *J Econ Entomol* **84**, 510-514 (1991).

11 Choe, D. H. & Rust, M. K. Horizontal transfer of insecticides in laboratory colonies of the Argentine ant (Hymenoptera : Formicidae). *J Econ Entomol* **101**, 1397-1405 (2008).
