# Supplementary material for: Declaration of local chemical eradication of the Argentine ant: Bayesian estimation with a multinomial-mixture model
Source: Sci Rep. 2017 Jun 13;7:3389. doi: 10.1038/s41598-017-03516-z (PMC5469785; doi:10.1038/s41598-017-03516-z)
Supplement: Supplementary file 3 — Program_code [file 41598_2017_3516_MOESM3_ESM.doc]

Prog.direct <- ########### set directry where "WinBUGS14" folder exist

direct <- ######### set data directry

require(R2WinBUGS)

Data <- read.csv(paste(direct, "data.csv", sep="")) # read observed data

# convert data frame into matrices

Data.spl <- split(Data, Data$point) # split into point unit

data.time <- NULL

data.num <- NULL

data.hold <- NULL

data.chem <- NULL

for (u in 1:(length(Data.spl))) {

data.time <- rbind(data.time, as.character(Data.spl[[u]]$time[1:59]))

# use data from the start to 59 th months

data.num <- rbind(data.num, Data.spl[[u]]$abundance[1:59])

data.hold <- rbind(data.hold, Data.spl[[u]]$hold[1:59])

data.chem <- rbind(data.chem, Data.spl[[u]]$chem[1:59])

}

####### Bayesian inference

dataB <- list(

Y.obs=data.num, # number of removals at each survey and each point

Y.capt=apply(data.num,1,sum), # total number of removals at each point

hold=data.hold, # trap status (1: hold, 0: lost)

chem=data.chem, # pesticide status (1: applied, 0: not applied)

npoint=nrow(data.num), # number of survey point

tcapt =ncol(data.hold)) # N. data

set.seed(1)

seed <- 1 # seed for WinBUGS

initB <- function() list(

p.d0=runif(1,0,1), p.c0=runif(1,0,1), shape=rlnorm(1), rate=rlnorm(1), lambda=rlnorm(nrow(data.num)), Y.liv=apply(data.num+1+rpois(length(data.num),1),1,sum) )

parmB <- c("p.d0", "p.c0",  "Y.liv", "Y.tokai", "Y.jonan",

"prob.tokai", "prob.jonan", "Y.suv")

modelB <- function() {

p.d0 ~ dunif(0,1)

p.c0 ~ dunif(0,1)

  shape ~ dgamma(1E-2, 1E-2)

rate ~ dgamma(1E-2, 1E-2)

          for (i in 1:npoint) { # loop for survey point

          for (t in 1:tcapt) { # loop for survey time

p.c[i,t] <- p.c0*hold[i,t] # Equation 6

p.d[i,t] <- p.d0*chem[i,t] # Equation 7

        }  # t loop end

########## joint probabilities for all survey times:

         for (t in 1:tcapt) {

log.suv[i,t] <- log(1 - p.c[i,t]) + log(1 - p.d[i,t])

# log of per capita survival probability

log(pr.suv[i,t]) <- sum(log.suv[i,1:t])

# log of joint probability of an individual's survival until t-th surveys

} # prd.suv[i,t] = ∏ (1 - p.d[i,k])(1 - p.c[i,k])

p.cap[i,1] <- (1-p.d[i,1]) * p.c[i,1] # t = 1

         for (t in 2:tcapt) { # t > 1

     p.cap[i,t] <- pr.suv[i,t-1] * (1 - p.d[i,t]) * p.c[i,t]

# joint probability of individual's being captured in the t-th survey

          } #  t loop end

     sum.p.cap[i] <- sum(p.cap[i,1:tcapt])

# probability of being captured throughout the surveys

          for (t in 1:tcapt) {

     mu.p.cap[i,t] <- p.cap[i,t] / sum.p.cap[i]

# probability of being captured at t-th survey among that of total captures

     }

     Y.obs[i,1:tcapt] ~ dmulti(mu.p.cap[i,1:tcapt], Y.capt[i])

# conditional multinomial observation model (Equation 1)

     Y.capt[i] ~ dbin(sum.p.cap[i], Y.liv[i])

# the number of captures out of the latent inhabitants (Equation 2)

     Y.liv[i] ~ dpois(lambda[i])

# latent inhabitants (random variable) exposed to the survey (Equation 3)

lambda[i] ~ dgamma(shape, rate) # negative binomial (Equation 3)

########## derieved posteriors ##########

for (t in 1:tcapt) {

Y.suv[i,t] ~ dbin(pr.suv[i,t], Y.liv[i])

# Number of survived individuals (Equation 4)

}

     }   # i loop end

for (t in 1 : tcapt) {

Y.tokai[t] <- sum(Y.suv[29:51,t])

Y.jonan[t] <- sum(Y.suv[1:28,t])

# Total number of survived individuals (Fig.1)

test.tokai[t] <- step(Y.tokai[t]-1)

test.jonan[t] <- step(Y.jonan[t]-1)

prob.tokai[t] <- mean(test.tokai[t])

prob.jonan[t] <- mean(test.jonan[t])

# Pr(N ≥ 1): probability of population persistence (not eradicated yet) (Fig. 2)

} # t loop end

}

modelpath <- file.path(tempdir(), "modelB.bug")

write.model(modelB, modelpath)

N.iter <- 220000 # set iteration

N.burn <- 20000 # set burnin

N.thin <- 100 # set thinning

# call WinBUGS from R

mcmc <- bugs(data=dataB, inits=initB, parameters=parmB, model.file=modelpath,

n.chains=3, n.iter=N.iter, n.burnin=N.burn, n.thin=N.thin, bugs.seed=seed,

bugs.directory=paste(Prog.direct, "WinBUGS14", sep=""))
